# Sex Difference in Cutoff and Prevalence of Sarcopenia among 300,090 Urban Korean Population: Association with Metabolic Syndrome

**DOI:** 10.3390/medicina58101361

**Published:** 2022-09-28

**Authors:** Chul-Hyun Park, Jong Geol Do, Yong-Taek Lee, Kyung Jae Yoon

**Affiliations:** 1 Department of Physical and Rehabilitation Medicine, Kangbuk Samsung Hospital, Sungkyunkwan University School of Medicine, Seoul 03181, Korea; 2 Department of Physical and Rehabilitation Medicine, Samsung Medical Center, Sungkyunkwan University School of Medicine, Seoul 06351, Korea; 3 Department of Health Sciences and Technology, SAIHST, Sungkyunkwan University, Seoul 06355, Korea

**Keywords:** sarcopenia, metabolic syndrome, threshold levels, sex difference

## Abstract

*Background and Objectives*: The study aimed to establish the threshold values and prevalence of sarcopenia and to investigate the association of sarcopenia with metabolic syndrome in an urban Korean population. *Materials and Methods:* The study included 300,090 adults who underwent anthropometric analyses by bioelectrical impedance analyzer. Sarcopenia was defined as: (1) class I, skeletal muscle mass index (SMI) within −1 to −2 standard deviations (SDs); (2) or class II, <−2 SD of SMI in a young population. *Results:* Low SMI threshold levels for class I and class II sarcopenia were 39.8 and 36.7% in men, and 35.5 and 32.3% in women. Among all age groups, the prevalence rates of sarcopenia were highest in the age group 80–89 years. Following adjustment for possible confounders including age, sex, height, metabolic and health behavioral factors, adjusted odds ratios (95% confidence intervals) for the risk of metabolic syndrome were 2.43 (2.33–2.54) for class I and 2.69 (2.49–2.91) for class II sarcopenia, compared with the normal reference. Sarcopenia was more strongly associated with metabolic syndrome in women than men (*p* for interaction < 0.01). The threshold values and prevalence of sarcopenia were demonstrated in a large Korean urban population. *Conclusions:* This study identified that sarcopenia was associated with increased risk of metabolic syndrome, showing itself to be significantly higher in women than men.

## 1. Introduction

Sarcopenia, described as a generalized loss of skeletal muscle mass, is an emerging health problem in an aging society [1,2]. Sarcopenia may impair quality of life and potentially lead to physical disability [3]. Recent studies demonstrated that sarcopenia was associated with chronic conditions such as cardiovascular diseases, diabetes mellitus, liver disease, chronic nephropathy, and metabolic disorders [4,5,6,7,8]. Furthermore, sarcopenia is strongly associated with frailty syndrome [9,10]. Because frailty is a state of depleted body reserves from multi-dimensional aspects such as physical ability, cognition, and health triggered by a stressor [11], sarcopenia appears to be one of the main components of clinical frailty syndrome associated with physical frailty [12]. Frailty syndrome increases with aging and is closely related to muscle weakness and dysfunction [13]. Therefore, sarcopenia can be regarded as a principal pathway of adverse outcomes of physical frailty [12]. Furthermore, the frailty syndrome is reported to be associated with cardiovascular disorders such as ischemic stroke, coronary artery disease and peripheral artery disease [14,15,16]. Therefore, measuring skeletal muscle mass is important to determine sarcopenia. Currently, dual-energy X-ray absorptiometry (DEXA) is a well-defined evaluation tool for measuring skeletal muscle mass [17,18]. However, DEXA has several limiting factors such as its relative expense, the need for a specialized radiologist, radiation exposure, and is a time-consuming and non-portable system that requires patients to visit a center [18]. Thus, although DEXA is a precise evaluation method for analyzing body composition, it is not a routine screening procedure for identifying low skeletal muscle mass [19]. Alternatively, bioelectrical impedance analysis (BIA) can quantify skeletal muscle mass rapidly and simply without radiation exposure or specialized staff. The test can be done at the patient bedside [19,20]. Furthermore, many studies reported that BIA has good validity and correlation with DEXA for measuring body composition in both middle-aged [21,22] and elderly participants [23,24]. Therefore, BIA is now regarded as a routine evaluation tool to analyze body composition especially in clinical settings for large-group screening in health programs.

Previous studies reported significant differences in cutoff values for sarcopenia among different ethnic populations due to lifestyle, body size, and cultural backgrounds [25,26]. Despite the clinical importance of sarcopenia, no broadly accepted threshold value exists for using BIA to determine low skeletal muscle to define sarcopenia status in Korean metropolitan residents. Several reports found that the proportions of sarcopenia were substantially higher not only in elderly but also in young- and middle-aged healthy subjects [4,26,27]. Since recent studies suggested that early diagnosis of sarcopenia is important for preventing adverse outcomes [28,29], detecting low muscle mass for sarcopenia could be an important health screening test to identify the risk of developing chronic conditions or metabolic diseases. Therefore, the establishment of ethnic-specific threshold values and prevalence of sarcopenia status is essential in the Korean population. Furthermore, the impact of sarcopenia status on public health conditions, such as metabolic syndrome, should be explored to reduce healthcare burdens and develop prevention-oriented health programs for an aging society.

Metabolic syndrome is a serious health condition characterized by the co-presence of several risk factors of cardiovascular diseases including increased blood pressure, hyperglycemia, dyslipidemia, and excess body fat around the waist [30]. A strong association between metabolic syndrome and oxidative stress has been found [31]. Enhanced oxidative stress is closely related to increased body mass index, blood pressure, and triglycerides [32,33]. Moreover, ‘obesity and insulin resistance’ are the components of metabolic syndrome, which are most strongly associated with metabolic syndrome [34]. Systemic oxidative stress plays a major role in resulting insulin resistance and hyperglycemia [31]. Furthermore, an elevation of carotid arterial stiffness and a risk of cardiovascular disease are closely associated with metabolic syndrome through a pathway of oxidative stress [33,35]. Because oxidative stress and cardiovascular diseases are known to be elevated in sarcopenia [36,37], metabolic syndrome under the condition of sarcopenia should be investigated.

The aims of our study were (1) to establish threshold values for low skeletal muscle mass identifying sarcopenia status using BIA, (2) to identify prevalence rates for sarcopenia in multiple age groups, and (3) to investigate the association between sarcopenia and metabolic syndrome in a large sample of Korean men and women.

## 2. Methods

### 2.1. Study Subjects

The Kangbuk Samsung Health Study used data from a cohort of Korean people who undertook comprehensive annual medical examinations at Kangbuk Samsung Hospital Total Healthcare Center in Seoul, South Korea [6,38]. The participants were inhabitants of either Seoul or Kyung-Gi province. The cohort used in this study was 300,090 men and women (aged 20–89) who were evaluated for body composition between January 2012 and December 2018. Ethics approval for the study protocol and data analysis was obtained from the Institutional Review Board of Kangbuk Samsung Hospital (KBSMC 2020-08-024) and was conducted in accordance with the 1975 Declaration of Helsinki. The Institutional Review Board exempted the requirements for informed consent because the researchers retrospectively assessed de-identified data for analytical purposes.

### 2.2. Laboratory and Anthropometric Measurements

Data on demographic characteristics, smoking status, alcohol history, degree of physical activity, and history of hypertension, hyperlipidemia, and diabetes mellitus were collected by examining physicians using standardized self-administered questionnaires. Individuals with smoking history were categorized into never, former, or current smokers. Individuals with alcohol consumption over 20 g/day were defined as a heavy drinker. Degree of physical activity was evaluated using the International Physical Activity Questionnaire Short Form. Regular physical activity was defined as vigorous exercise more than 3 times/week for >20 min per session or moderate exercise more than 5 times/week for >30 min per session.

Blood specimens were obtained from the antecubital vein after an overnight fast of at least 10 h. Serum biochemical parameters such as total cholesterol, triglycerides, high-density lipoprotein cholesterol, low-density lipoprotein cholesterol, fasting glucose, creatinine, alanine aminotransferase (ALT), high sensitivity C-reactive protein (hs-CRP), and total vitamin D were measured using an auto-analyzer (747 Automatic Analyzer, Hitachi, Tokyo, Japan).

Anthropometric measurements of height, body weight, waist circumference, and body composition were evaluated by trained nurses. Waist circumference was estimated as the lowest abdomen circumference between the umbilicus and the xyphoid process. Skeletal muscle mass (kg) was evaluated using a multi-frequency BIA with eight-point tactile electrodes (InBody 720, Biospace Co., Seoul, Korea). The BIA was calibrated every morning before tests and validated for accuracy and reproducibility for analyzing body composition. For adjusting the effect of body weight, skeletal muscle mass index (SMI) was estimated as: SMI in % = skeletal muscle mass (kg)/weight (kg) × 100, based on the established method by Janssen et al. [3].

### 2.3. Sarcopenia Status and Metabolic Syndrome

The distribution of SMI for young men and women aged 20–39 was used as the reference group (82,537 men and 80,529 women) to establish threshold levels of low muscle mass for defining sarcopenia. Definition of sarcopenia was based on the method developed by Janssen et al. using the bioelectrical impedance data [3]. The cutoff points of the sarcopenia were determined by −1 SD and −2 SD in the young reference group, analogous to the osteoporosis definition. This definition of sarcopenia has been used in many previous studies [3,39,40,41]. Class I sarcopenia was defined as SMI within –1 SD to –2 SD of the sex-specific mean value of SMI in the young reference group. Class II sarcopenia was defined as less than −2 SD from the sex-specific mean value of SMI in the young reference group. SMI greater than −1 SD of the sex-specific mean value was considered as the normal range of SMI.

Metabolic syndrome criteria used in this study were defined by the Adults Treatment Panel III (ATP III) with adjusted body mass index (BMI) and waist circumference for Asians [42,43]. In Asian populations, the reduced value of BMI is ≥25 kg/m^2^ for both sexes and waist circumference is ≥90 cm (men) and ≥80 cm (women) as suggested by revised Asian-Pacific Criteria from the World Health Organization (WHO) Western Pacific Region [43].

### 2.4. Statistical Analysis

Continuous variables that followed a normal distribution were analyzed with Student’s t-test or and described as the mean ± SD. Continuous variables that did not follow a normal distribution (skewed) were analyzed with the Mann–Whitney U test and expressed as the median (interquartile range) such as triglycerides, fasting glucose, creatinine, and hs-CRP. Categorical variables as number with percent and analyzed by chi-square or Fisher’s exact tests.

One-way analysis of variance (ANOVA) test was used to compare the means of the SMI according to age groups of 20–29, 30–39, 40–49, 50–59, 60–69, 70–79, and 80–89 years with a linear trend. After one-way ANOVA test, post hoc analysis was performed by Bonferroni method for the group comparisons. Sex-specific prevalence rates of sarcopenia in age groups of 20–29, 30–39, 40–49, 50–59, 60–69, 70–79, and 80–89 years were calculated for healthy Korean adults based on threshold values for class I and class II sarcopenia from the young reference group.

Multivariate logistic regression analyses were conducted to determine the associations between sarcopenia status and presence of metabolic syndrome. Odds ratios (ORs) were calculated as risks for presence of metabolic syndrome in class I sarcopenia and class II sarcopenia compared with normal body composition (reference). We used two models with multivariate adjustments for possible confounding variables. The first model (model 1) was adjusted for age, sex, height, serum creatinine, ALT, hs-CRP, and total vitamin D. The second model (model 2) was additionally adjusted for health-related behavioral factors such as current smoker, heavy drinking, and regular physical activity.

Stratified analyses were performed in prespecified subgroups defined by sex (men vs. women). Interactions between subgroups were analyzed using likelihood ratio tests comparing models with and without multiplicative interaction terms. All statistical analyses were used IBM SPSS version 24.0 for windows. *p* values below 0.05 were considered statistically significant.

## 3. Results

### 3.1. Baseline Characteristics and SMI Values of Study Participants

**Figure 1:**
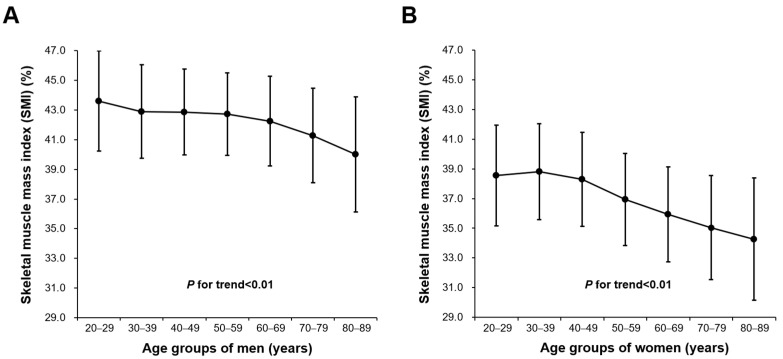
Means and standard deviations for SMI across age groups (20–29, 30–39, 40–49, 50–59, 60–69, 70–79, 80–89 years) for (**A**) men and (**B**) women. SMI = skeletal muscle mass index.

Baseline characteristics and SMI values for all age groups in urban Korean men and women are summarized in Table 1. Means (SD) for age were 40.3 (9.4) years for men and 39.4 (10.2) years for women and for SMI were 42.8 (3.0)% for men and 38.2 (3.4)% for women (*p* < 0.01). There was a significant linear trend between lowering SMI and increased age groups in men and women, respectively, (*p* for linear trend < 0.01; Figure 1) showing the lowest mean values of SMI in 80–89 year in men and women (men, 40.0 ± 3.9; women, 34.2 ± 4.1). In Bonferroni post hoc analysis for group comparisons, the SMI of the 80–89 year group was significantly lower than those in age groups of 20–29, 30–39, 40–49, 50–59, 60–69, and 70–79 year, respectively, in men (all post hoc *p* < 0.01). In women, the SMI of the 80–89 year group was significantly lower than that in the age groups of 20–29, 30–39, 40–49, 50–59, and 60–69 year, respectively, (all post hoc *p* < 0.01). However, the SMI of the 80–89 year group was similar to that of the 70–79 year group (post hoc *p* = 0.99).

### 3.2. Threshold Levels for Class I and Class II Sarcopenia Based on Young Reference Group

**Figure 2:**
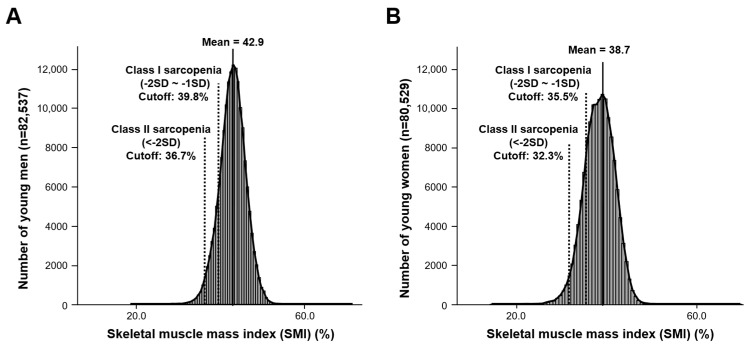
Number of (**A**) young men and (**B**) young women according to SMI and threshold levels for class I and class II sarcopenia. SMI, skeletal muscle mass index. SMI = skeletal muscle mass index.

For determination of the threshold levels of low SMI for class I and class II sarcopenia, the means of the SMI of young men and women (men 82,537; women 80,529) were estimated as 42.9 (3.1)% and 38.7 (3.2)%, respectively (Table 2). SMI threshold levels were 39.8% for class I sarcopenia and 36.7% for class II in Korean men (Figure 2A). In women, SMI threshold levels were 35.5% for class I sarcopenia and 32.3% for class II (Figure 2B).

### 3.3. Prevalence Rate of Sarcopenia by Age Group

**Figure 3:**
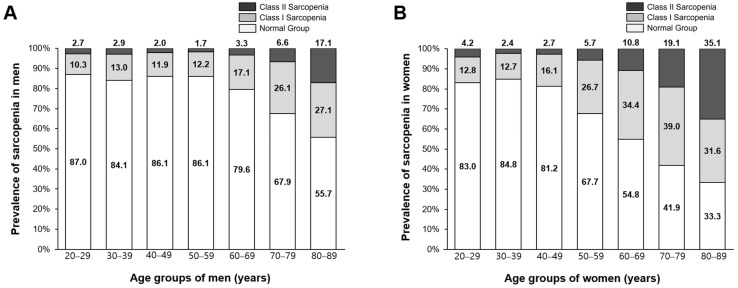
Prevalence rates for sarcopenia status across age groups (20–29, 30–39, 40–49, 50–59, 60–69, 70–79, 80–89 years) for (**A**) men and (**B**) women. SMI = skeletal muscle mass index.

Based on the threshold values for identifying sarcopenia, prevalence rates were estimated for Korean men and women by age group (Figure 3). For all groups, prevalence rates for class II sarcopenia were highest in the age group 80–89 years in both sexes. The prevalence rate of class II sarcopenia in the age group 80–89 years was higher for women than men (women 35.1% vs. men 17.1%; *p* < 0.01). For the group aged 70–79, the prevalence rates for class II sarcopenia were 6.6% for men and 19.1% for women (*p* < 0.01). For the age group 60–69 years, the prevalence rate for class II sarcopenia was higher for women than men (10.8% vs. 3.3%, respectively; *p* < 0.01).

### 3.4. Sarcopenia Status and Metabolic Syndrome

Proportions of metabolic syndrome by groups were 6.9% for normal body composition, 23.2% for class I and 35.2% for class II sarcopenia (*p* < 0.01). Results of multivariate logistic regression analyses to assess associations of metabolic syndrome with class I and class II sarcopenia are in Table 3. Model 1 adjusted for possible confounding factors of age, sex, height, serum creatinine, ALT, hs-CRP, and total vitamin D. Individuals with class I and class II sarcopenia exhibited a significantly increased risk of metabolic syndrome (adjusted ORs [95% CI] compared with normal reference; class I, 2.42 [2.33–2.52]; class II, 2.62 [2.45–2.80]). Model 2 was additionally adjusted for health behavioral factors (i.e., current smoker, heavy drinking, and regular physical activity). Adjusted ORs (95% CI) for risk of metabolic syndrome were 2.43 (2.33–2.54) for class I and 2.69 (2.49–2.91) for class II sarcopenia compared with the reference.

### 3.5. Sex Differences in the Association between Sarcopenia and Metabolic Syndrome

**Figure 4:**
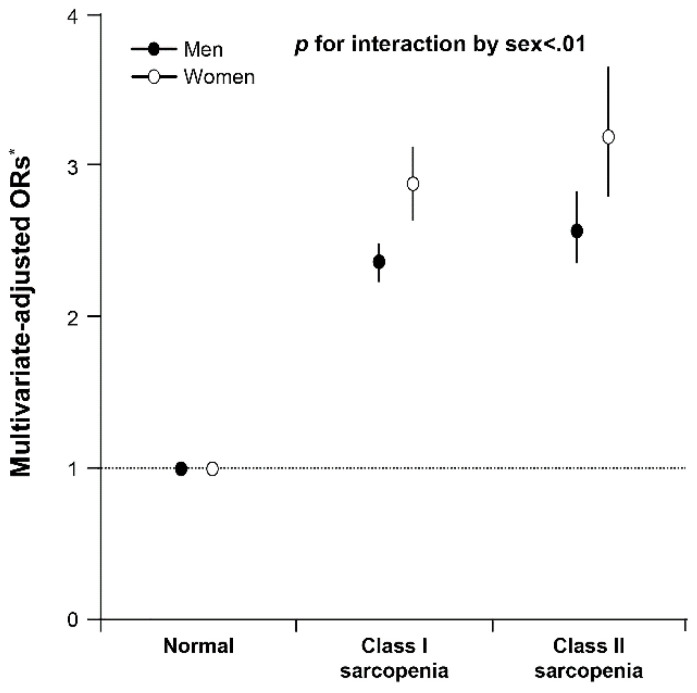
Multivariate-adjusted ORs (95% CI) for presence of metabolic syndrome according to sarcopenia status in men and women. *p* value for interaction by sex between metabolic syndrome and sarcopenia status is indicated. Adjusted ORs were estimated from a multivariate logistic regression model with presence of metabolic syndrome as the outcome. * Multivariate model 2 was adjusted for age, height, serum creatinine, ALT, hs-CRP, total vitamin D, current smoker, heavy drinking, and regular physical activity. ALT = alanine aminotransferase; CI = confidence interval; hs-CRP = high-sensitivity C-reactive protein; ORs = odds ratios.

Based on subgroup analyses for men and women, the interaction between sarcopenia status and sex for metabolic syndrome was significant (*p* for interaction < 0.01; Figure 4, Table 3). In Model 2 multivariate analyses, sarcopenia status was more strongly associated with risk of metabolic syndrome in women than in men (adjusted ORs [95% CI]; 3.17 [2.76–3.64] vs. 2.57 [2.34–2.82]).

## 4. Discussion

This study presented threshold values for low SMI identifying sarcopenia status and the prevalence rates for multiple age groups in a large Korean population. Additionally, we demonstrated that subjects with sarcopenia status based on these thresholds had a higher risk of metabolic syndrome than those with normal muscle mass. Sarcopenia status was more strongly associated with metabolic syndrome in women than in men.

Although the threshold level for determining the low SMI in sarcopenia is a well-accepted method, reported threshold levels for low SMI differ by sex, ethnicity, and race [2,26,44]. Therefore, the European Working Group on Sarcopenia in Older People (EWGSOP) and many previous studies suggest that cutoff levels for low SMI to determine sarcopenia should be referenced based on specific nations and countries [45]. Furthermore, SMI equations should be considered using either weight-adjusted or height-adjusted SMI, which is still in consensus discussions [45]. Many previous reports on sarcopenia use skeletal muscle mass divided by height squared [46,47,48]. However, recent studies found that height-adjusted SMI could severely underestimate the prevalence rate of sarcopenia in Southeast Asians including Chinese and Korean populations [49,50,51]. In particular, the height-adjusted equation can underrate sarcopenia in overweight and obese adults. Lim et al. demonstrated that sarcopenic obesity determined by weight-adjusted SMI is more closely associated with metabolic syndrome than height-adjusted SMI in a longitudinal study [52]. Therefore, our study used the weight-adjusted equation to determine low SMI for sarcopenia and estimated the prevalence rate of sarcopenia status in the Korean population.

The present study used the BIA to measure SMI rather than DEXA, an important consideration when applying threshold levels of low SMI to the elderly subjects. The BIA is a commonly used method for estimating body composition and has many advantages over DEXA [19,20]. BIA uses a safe portable device that does not involve radiation exposure. Moreover, BIA can be applied for the health screening of a large population because of its speed and simplicity with no need for a specialized radiologist [19,21]. Although DEXA is well-validated technique for analyzing body composition at the molecular level, recent studies show that BIA is well correlated with DEXA [21,53]. Furthermore, BIA has significant correlations with precise imaging techniques such as computed tomography. Therefore, many studies and working groups utilized BIA as a diagnostic device for detecting low skeletal muscle mass for determination of sarcopenia as a reliable alternative to DEXA [19].

In the present study using BIA, threshold levels for class I and class II sarcopenia were set as 39.8% for men and 35.5% for women for class I, and 36.7% for men and 32.3% for women for class II sarcopenia, respectively. Two previous studies from Turkey and United States are available that used BIA [3,40]. Bahat et al. [40], in a study in Turkey, demonstrated threshold levels of 40.4% for men and 37.2% for women for class I, and 37.4% for men and 33.6% for women for class II sarcopenia. These levels were slightly higher than in those of our study (Table 4). On the other hand, Janssen et al. [3] in the US study found cutoff points of 37.0% for men and 28.0% for women for class I, and 31.0% for men and 22.0% for women for class II sarcopenia, respectively. These rates were somewhat lower than those for the Korean population. Compared with the threshold values from our study, differences were higher for the US study than the Turkish study [40]. The reason for the difference could be that the United States has a variety of different ethnic groups that are considerably different from the relatively homogenous ethnic group of Koreans [54,55]. In contrast, the Turkish ethnic groups originate from Central Asia and Western Asia, which is regionally closed to South Korea located in East Asia [56]. Therefore, the establishment of ethnic or nation-specific cutoff values for identifying sarcopenia is important, especially for clinical application using BIA, as demonstrated in this study in Korea adults.

An important observation of this study was that the people with sarcopenia had a higher rate of metabolic syndrome than people with normal muscle mass. After adjusting for possible confounding factors of age, sex, height, and metabolic and health-related behavioral factors, risk of metabolic syndrome with class I sarcopenia was 2.43-fold increased and risk with class II sarcopenia was 2.69-fold higher compared with the reference, respectively. Lu et al. [57] showed that a sarcopenia group had a 1.98-fold higher risk of metabolic syndrome compared to a normal group, which was consistent with our data. However, the OR for risk of metabolic syndrome in the previous study was slightly lower than our results. There could be a several explanations. First, the previous study had a small number of participants, at 600 subjects, compared with the present study of 300,090 participants. Second, we added various possible confounding factors related to skeletal muscle mass in the analysis of associations of metabolic syndrome with sarcopenia. The previous study only used age, sex and health-related behaviors as confounders. However, we included the health-related behavioral factors of smoking, alcohol consumption, physical activity; and important metabolic factors of serum hs-CRP, creatinine, ALT, and total vitamin D, which showed significant associations with sarcopenia in previous studies [58,59,60,61]. Therefore, our finding that sarcopenia status was a magnifying risk factor for metabolic syndrome is valuable information to be considered in managing public health.

Interestingly, the current study demonstrated that sarcopenia status had stronger associations with metabolic syndrome in women than in men. The association persisted after adjusting for possible confounding variables, which supported the significance of our study. Sex-specific differences could be discussed by a few possible explanations. First, the distribution and fiber type of skeletal muscle is different in men and women, which may affect risk of metabolic syndrome [62,63]. Mesinovic et al. demonstrated that muscle function and quality are related to metabolic syndrome [64]. Thus, different muscle types and quality could affect sex differences in the association between metabolic syndrome and sarcopenia. Second, differences in subclinical inflammation status between sexes may mediate the association of sarcopenia with metabolic syndrome. Previous studies report that associations of serum hs-CRP with obesity and sarcopenic obesity are stronger in women than in men [61,65], which were in line with our study. To the best of our knowledge, our study is the first to report the role of sex differences in associations between sarcopenia and metabolic syndrome in a large cohort of adults.

The present study primarily reports weight-adjusted threshold values for class I and class II sarcopenia with prevalence rates for multiple age groups in a large population-based study. These results could be used as representative threshold values for low SMI in Korean urban residents. The prevalence rates of sarcopenia in men and women increased overall as age increased. Interestingly, the mean SMI in women in their 20s was lower than in women in their 30s. The prevalence rate for class II sarcopenia was higher in women in their 20s than in women in their 30s. The reason could be the cultural background of South Korea. Korean women in their 20s try to lose weight through intense diet even if they are average weight, with the result of being underweight with decreased skeletal muscle mass [66,67]. Another finding of our study was that the prevalence rate for class II sarcopenia of men in their 60s was twice as high as men in their 50s (3.3% vs. 1.7%). However, in Korean women, the prevalence rate for class II sarcopenia doubled in their 50s compared to women in their 40s. Messiera et al. found that menopause leads to decreased SMI, suggesting that the prevalence rate of sarcopenia increases at a time when significant changes in hormonal status, such as estrogen level, occur [68]. Therefore, low SMI for sarcopenia status tended to happen earlier in women than in men.

Our study has several limitations. First, the middle-aged population was most represented among the study subjects. However, we addressed this issue by analyzing data by multiple age groups. Second, skeletal muscle mass was measured only using BIA despite the dominance of DEXA for estimation of muscle mass for research. However, the validity and reproducibility of the device used in our study was verified [69,70] and correlates well with DEXA results [21,53,71]. Furthermore, BIA examinations were performed after fasting for more than 8 h, minimizing the effect of hydration.

In conclusion, we presented thresholds of low SMI for the identification of sarcopenia status and prevalence rates for multiple age groups in a large group of Korean adults. This could be valuable information for medical health screening or clinical purposes using BIA. Furthermore, we demonstrated that sarcopenia status was an independent risk factor for metabolic syndrome, showing an especially strong association for women compared to men. Therefore, SMI values below thresholds could help clinicians identify people who are at risk for metabolic and functional impairment.

## Figures and Tables

**Table 1:** Basic characteristics and age distribution of men and women in study subjects (*n* = 300,090).

Characteristics	Men (n = 160,664)	Women (n = 139,426)	*p* Value
Age (years)	40.3 (9.4)	39.4 (10.2)	<0.01
Height (cm)	173.1 (5.8)	160.3 (5.3)	<0.01
Weight (kg)	73.4 (10.2)	56.1 (8.2)	<0.01
Body mass index (kg/m^2^)	24.4 (2.9)	21.8 (3.1)	<0.01
Waist circumference (cm)	86.2 (7.9)	76.2 (8.3)	<0.01
Fat mass (kg)	17.2 (6.0)	17.0 (5.7)	<0.01
Skeletal muscle mass (kg)	31.4 (3.6)	21.3 (2.3)	<0.01
SMI (%) ^1^	42.8 (3.0)	38.2 (3.4)	<0.01
SBP (mmHg)	113.2 (11.5)	100.9 (11.6)	<0.01
DBP (mmHg)	73.9 (9.4)	64.9 (8.7)	<0.01
Total cholesterol (mg/dL)	199.3 (34.5)	188.4 (33.0)	<0.01
Triglycerides (mg/dL)	114 (81–164)	71 (54–98)	<0.01
LDL-C (mg/dL)	126.8 (31.4)	110.9 (30.2)	<0.01
HDL-C (mg/dL)	52.6 (12.8)	64.5 (14.8)	<0.01
Fasting glucose (IU/L)	95 (90–102)	91 (86–96)	<0.01
Creatinine (mg/dL)	1.0 (0.9–1.1)	0.7 (0.6–0.8)	<0.01
ALT (U/L)	29.1 (24.1)	15.8 (14.3)	<0.01
hs-CRP (mg/L)	0.05 (0.03–0.10)	0.03 (0.02–0.07)	<0.01
Total vitamin D (nmol/L)	17.6 (6.7)	14.2 (6.6)	<0.01
Hypertension (%)	12.4	5.8	<0.01
Hyperlipidemia (%)	17.7	8.5	<0.01
Diabetes mellitus (%)	4.0	1.7	<0.01
Current smoker (%)	37.0	2.6	<0.01
Heavy drinking (%)	28.5	3.8	<0.01
Regular physical activity (%)	14.8	12.6	<0.01
Age subgroups (years)			
20–29 (*n*)	10,359	15,858	
30–39 (*n*)	72,178	64,671	
40–49 (*n*)	53,269	37,427	
50–59 (*n*)	17,671	14,359	
60–69 (*n*)	5792	5800	
70–79 (*n*)	1325	1254	
≥80 (*n*)	70	57	

Data are expressed as mean (standard deviation) or median (interquartile range). ALT = alanine aminotransferase; DBP = diastolic blood pressure; HDL-C = high-density lipoprotein cholesterol; hs-CRP = high sensitivity C-reactive protein; LDL-C = low-density lipoprotein cholesterol; SBP = systolic blood pressure; SMI = skeletal muscle mass index. ^1^ SMI (skeletal muscle mass index) (%) = skeletal muscle mass (kg)/weight (kg) × 100.

**Table 2:** Cutoff levels of class I and class II sarcopenia in study subjects.

	Young Men	Young Women
No. of participants (n)	82,537	80,529
Mean value of SMI (%)	42.9 (3.1)	38.7 (3.2)
SMI cutoff levels (%) of sarcopenia		
Class I sarcopenia	39.8	35.5
Class II sarcopenia	36.7	32.3

Data are presented as mean (standard deviation), percentage or number. SMI = skeletal muscle mass index.

**Table 3:** Adjusted ORs (95% CI) of metabolic syndrome according to sarcopenia status in study subjects.

	Age Adjusted OR	Model 1	Model 2
All			
Normal body composition	1 (reference)	1 (reference)	1 (reference)
Class I sarcopenia	3.76 (3.66–3.86)	2.42 (2.33–2.52)	2.43 (2.33–2.54)
Class II sarcopenia	6.82 (6.51–7.15)	2.62 (2.45–2.80)	2.69 (2.49–2.91)
Men			
Normal body composition	1 (reference)	1 (reference)	1 (reference)
Class I sarcopenia	4.20 (4.06–4.35)	3.48 (3.33–3.64)	2.36 (2.25–2.49)
Class II sarcopenia	7.39 (6.93–7.87)	5.36 (4.96–5.80)	2.57 (2.34–2.82)
Women			
Normal body composition	1 (reference)	1 (reference)	1 (reference)
Class I sarcopenia	4.46 (4.23–4.69)	4.56 (4.26–4.88)	2.87 (2.62–3.16)
Class II sarcopenia	8.76 (8.12–9.45)	8.55 (7.74–9.45)	3.17 (2.76–3.64)

CI = confidence interval; Model 1: Adjustment for age, sex, height, serum creatinine, ALT, hs-CRP, and total vitamin D. Model 2: Model 1 + current smoker, heavy drinking, regular physical activity.

**Table 4:** Comparative data for threshold levels of low skeletal muscle mass for class I and class II sarcopenia in different populations.

	Men	Women
Present study		
Class I	39.8	35.5
Class II	36.7	32.3
Janssen et al. [3] from US		
Class I	37.0	28.0
Class II	31.0	22.0
Bahat et al. [40] from Turkey		
Class I	40.4	37.2
Class II	37.4	33.6

Data are presented as the skeletal muscle mass index (percentage).

## Data Availability

Data can be obtained from corresponding author upon reasonable request.
